# Mendelian randomization study of telomere length and bone mineral density

**DOI:** 10.18632/aging.202197

**Published:** 2020-12-15

**Authors:** Fashuai Wu, Yu Huang, Jialu Hu, Zengwu Shao

**Affiliations:** 1 Department of Orthopaedics, Union Hospital, Tongji Medical College, Huazhong University of Science and Technology, Wuhan 430022, China; 2 Department of Otorhinolaryngology, The Third Hospital of Wuhan, Wuhan 430070, China; 3 School of Computer Science, Northwestern Polytechnical University, Xi’an 710072, China

**Keywords:** two-sample Mendelian randomization, telomere length, bone mineral density, osteoporosis

## Abstract

Purpose: Some epidemiological studies and animal studies have reported a relationship between leukocyte telomere length (LTL) and bone mineral density (BMD). However, the causality underlying the purported relationship has not been determined. Here we performed a two-sample MR analysis to test the causal link between telomere length and BMD.

Results: Our research suggested no causal link of LTL and BMD using IVW method. The weighted median, MR-Egger regression and MR.RAPS method yielded a similar pattern of effects. MR-Egger intercept test demonstrated our results were not influenced by pleiotropy. Heterogeneities among the genetic variants on heel estimated BMD and TB-BMD vanished after excluding rs6028466. “Leave-one-out” sensitivity analysis confirmed the stability of our results.

Conclusion: Our MR analysis did not support causal effect of telomere length on BMD.

Methods: We utilized 5 independent SNPs robustly associated with LTL as instrument variables. The outcome results were obtained from GWAS summary data of BMD. The two-sample MR analysis was conducted using IVW, weighted median, MR-Egger regression and MR.RAPS method. MR-Egger intercept test, Cochran’s Q test and I^2^ statistics and “leave-one-out” sensitivity analysis were performed to evaluate the horizontal pleiotropy, heterogeneities and stability of these genetic variants on BMD.

## INTRODUCTION

The incidence of aging-related disorders has dramatically increased in modern society, owing to the development of healthcare and the changes of socio-economy and lifestyle [1]. Osteoporosis, which is clinically diagnosed largely through measurement of bone mineral density (BMD) examined by dual-energy X-ray absorptiometry (DXA), is a common aging-related systemic skeletal disease, characterized by low bone mass, micro-architectural deterioration of bone tissue and an increased risk to fracture [1–3]. In the United States, the prevalence of osteoporosis is estimated to increase to more than 14 million people in 2020, and the burden is projected to increase to over 3 million fractures and $25.3 billion each year by 2025 [4]. The etiology of osteoporosis is not well understood. It is well recognized that increasing age, female gender and a wide range of both environmental factors and genetic factors are associated with the disease [3, 5–7]. Traditional observational studies reported that the potential risk factors, including glucocorticoid therapy, low body mass index (BMI), physical inactivity, smoking, heavy alcohol consumption, inflammatory bowel disease and calcium and vitamin D deficiency etc., are related to BMD and fractures [8, 9].

Telomeres, capping and protecting the termini of eukaryotic chromosomes from fusion and degradation, are the dynamic nucleoprotein-DNA complexes, which consist of hexameric nucleotide sequences (TTAGGG) repeats and associated protective proteins [10, 11]. It is important for chromosomal stability and cellular integrity. Telomeres are shortened progressively during each cell division. Thus, they are recognized as a physiological marker of organism’s age, which will finally cause chromosomal instability, cellular senescence, and eventually cell cycle arrest and apoptosis [12, 13]. Recent studies in the understanding of human disease processes have shown the roles of telomere biology in the diseases of human aging and in some aging-related processes [11, 14]. Alterations of telomere length and telomere dysfunction have been linked to a wide range of diseases, including cancer, cardiovascular diseases, type 2 diabetes mellitus (T2DM) and neurodegenerative diseases [15–17]. Many findings that associate the alterations of telomere length as well as telomere dysfunction with age-related impaired homeostasis of bone cells which promote osteoporosis, have been reported. In the model of Wrn-/- Terc-/- mutant mice, the low bone mass phenotype and age-related osteoporosis are the result of dysfunctional telomeres that impaired osteoblast differentiation through accelerating cell senescence of bone-forming cells and their precursors [18]. In humans, premature aging syndromes including dyskeratosis congenita and Werner syndrome which are characterized by telomere dysfunction commonly occur with osteoporosis [19, 20]. Telomere length is often approximated using the readily accessible leukocyte telomere length (LTL), as it is highly correlated with telomere length in other tissues and can be easily extracted from blood [21]. Based on these findings, it has been hypothesized that telomere length should be associated with BMD and osteoporosis. Valdes et al. showed that, in women, shortened LTL was independently correlated with low BMD in spine and forearm and the presence of osteoporosis [22]. In Chinese women aged 60–65 years, short LTL was associated with low BMD at femoral neck and high osteoporotic risk [23]. In a small prospective observational study in elderly men (age range 71–86 years), telomere length of peripheral blood leukocytes correlated with age-associated bone loss at different distal forearm sites [24]. However, several other observational human studies did not show the statistically significant associations between LTL and osteoporosis [25, 26]. Furthermore, in vitro studies with human trabecular osteoblasts and the measurement of LTL from osteoporotic women and age matched control subjects did not support the notion of the occurrence of a generalized premature cellular aging and accelerated telomere shortening in osteoporotic patients [27]. The studies, which had drawn inconsistent conclusions, were either based on limited samples or only explored the correlations between LTL and BMD and osteoporosis, and the epidemiological observational studies might be subjected to confounding factors and reverse causality [28]. A study, like randomized controlled trial (RCT), directly inferring the causal relationship of LTL and BMD and osteoporosis is helpful in recognizing the etiology of disease processes and identifying potential treatment strategies. However, RCTs are difficult or impractical to perform for its expensive, labor resource-intensive, time consuming and ethical limitations. As an alternative, Mendelian randomization (MR), mimic the design of RCT, is a popular yet more convenient technique to test the causality between an exposure (telomere length) and an outcome (BMD or osteoporosis) [28].

Mendelian randomization is a technique, using germline genetic variants as instrument variables (IV) for exposure to study the causal relation between the exposure phenotype and the outcome phenotype. In order to obtain unbiased estimates, MR need to fulfill three key assumptions: IV1) genetic variants used in analysis should be significantly associated with the exposure; IV2) genetic variants extracted as instrument variables for exposure are independent of confounding factors that are associated with the selected exposure and outcome; and IV3) the genetic variants affects the outcome only through the exposure and not via other biological pathways (i.e., no horizontal pleiotropic effect) Supplementary Figure 1 [29]. The aim of our study is to assess the causal link between telomere length and BMD under a two-sample MR study framework, in which we will use the summary statistics from genome-wide association study (GWAS) data of LTL and BMD.

## RESULTS

### Selection of instrumental variables

We selected 16 SNPs as instrumental variables to investigate causal relationships between LTL and BMD in European ancestry [30]. After performing the clumping process in which amongst those pairs of SNPs that had LD R-square above the specified threshold (R-square = 0.001), only the SNP with the lower P-value (P value retrieved from summary data of Mangino et al. [31]) would be retained, namely 10 independent SNPs were left as potential IVs for LTL. Querying these 10 LTL associated SNPs in the Phenoscanner database, we found rs6772228 (PXK), rs10936599 (TERC), rs2736100 (TERT) and rs755017 (ZBTB46) were significantly associated with phenotypes (rheumatoid arthritis, celiac disease, mean corpuscular hemoglobin, red blood cell count or body fat percentage) which were risk factors for osteoporosis or low BMD after Bonferroni correction (p < 0.05/10 = 0.005). The four SNPs were excluded. Further, based on the assumption that the genetic variant is independent of the outcome conditional on the exposure and confounders, rs11125529 (ACYP2) could be excluded for its significant association with heel bone mineral density (P = 4.41e-13). Eventually, 5 SNPs: rs7675998 (NAF1), rs9420907 (OBFC1), rs3027234 (CTC1), rs412658 (ZNF676), rs6028466 (DHX35) were included as IVs for LTL in further analyses. F statistic for the instrument-exposure association was 24.19, which was much greater than 10, demonstrating the tiny possibility of weak instrumental variables bias. Characteristics of SNPs predictive of the LTL were shown in Table 1.

**Table 1:** Characteristics of SNPs predictive of the leukocyte telomere length (LTL).

**SNP**	**Chr**	**Nearby Gene**	**EA**	**OA**	**EAF**	**β**	**Se**	**P value^1^**	**P value^2^**	**Sample size**	**removed**
rs7675998	4	NAF1	G	A	0.80	0.048	0.012	1.00e-2	4.35e-16	9161	No
rs9420907	10	OBFC1	C	A	0.14	0.142	0.014	1.14e-11	7.00e-11	9190	No
rs3027234	17	CTC1	C	T	0.83	0.103	0.012	2.75e-8	2.00e-8	9108	No
rs412658	19	ZNF676	T	C	0.35	0.086	0.010	1.83e-8	1.00e-8	9156	No
rs6028466	20	DHX35	A	G	0.17	0.058	0.013	4.00e-3	2.57e-8	9190	No
rs6772228	3	PXK	T	A	0.87	0.041	0.014	4.97e-2	3.91e-10	8630	Yes
rs10936599	3	TERC	C	T	0.76	0.100	0.011	1.76e-9	3.00e-31	9190	Yes
rs2736100	5	TERT	C	A	0.52	0.085	0.013	2.14e-5	4.38e-19	5756	Yes
rs755017	20	ZBTB46	G	A	0.17	0.019	0.0129	3.40e-1	6.71e-9	8026	Yes
rs11125529	2	ACYP2	A	C	0.16	0.065	0.012	6.06e-3	8.00e-10	9177	Yes
rs12696304	3	TERC	C	G	0.74	0.090	0.011	5.41e-8	4.00e-14	9012	Yes
rs1317082	3	TERC	A	G	0.71	0.097	0.011	4.57e-9	1.00e-8	9176	Yes
rs10936601	3	TERC	C	T	0.76	0.100	0.011	1.76e-9	4.00e-15	9190	Yes
rs9419958	10	OBFC1	T	C	0.13	0.129	0.013	5.26e-11	9.00e-11	9190	Yes
rs4387287	10	OBFC1	A	C	0.14	0.120	0.013	1.40e-9	2.00e-11	8541	Yes
rs8105767	19	ZNF208	G	A	0.25	0.064	0.011	1.00e-3	1.11e-9	9096	Yes

SNP: single-nucleotide polymorphism; Chr: chromosome; EA: effect allele; OA: other allele; EAF: Effect allele frequency; β: standard deviation change in leukocyte telomere length per copy of the effect allele; Se: standard error; P value^1^: P value from summary data of Mangino et al.[33]; P value^2^: P value from original study reports curated by the GWAS catalog.

### Two-sample Mendelian analysis for causal link of leukocyte telomere length with BMDs

We chose 5 independent SNPs associated with LTL in European ancestry to perform the MR analysis for the causal link between LTL and FN-BMD, LS-BMD, FA-BMD, heel estimated BMD, TB-BMD and TB-BMD (age over 60). The effect of all the 5 SNPs on the outcome GWAS was present, and no palindromic SNPs was found. Among these SNPs, only rs6028466 was nominally associated with reduced level of FN-BMD, heel estimated BMD and TB-BMD (FN-BMD: P=0.042, heel estimated BMD: P=0.007 and TB-BMD: P=0.025 respectively). None of the 5 SNPs was significantly associated with BMD outcomes at the Bonferroni-corrected significance threshold (p < 0.002) (i.e., 0.05/25), as shown in Supplementary Table 1.

**Figure 1:**
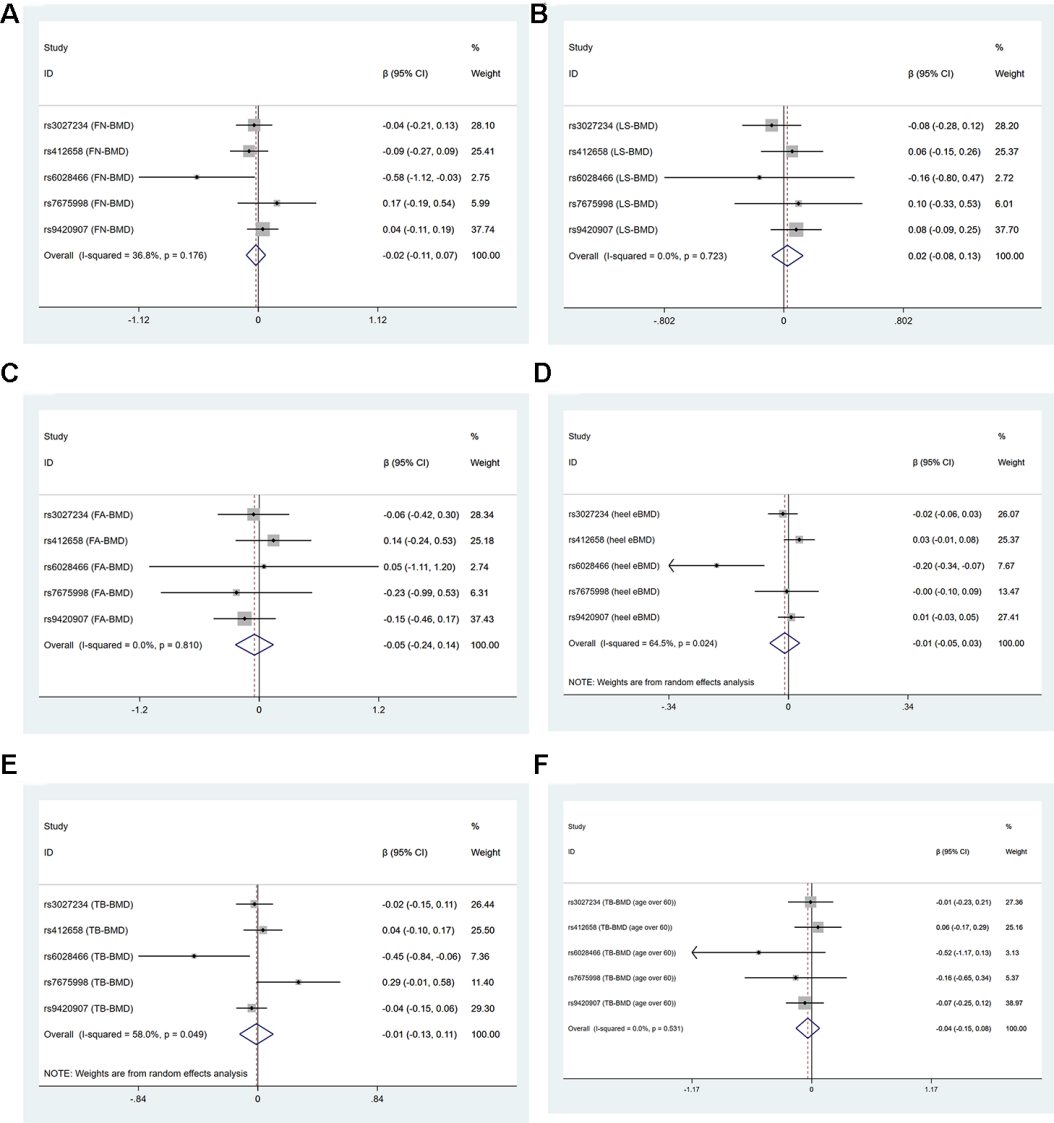
Forest plots for Mendelian randomization estimates of the association of leukocyte telomere length on BMDs (IVW method) (**A**) FN-BMD (**B**) LS-BMD (**C**) FA-BMD (**D**) heel estimated BMD (**E**) TB-BMD and (**F**) TB-BMD (age over 60).

In two-sample MR analysis, the decrease of LTL did not have a causal link with the level of FN-BMD, LS-BMD, FA-BMD, heel estimated BMD, TB-BMD and TB-BMD (age over 60) basing on IVW, WM, MR-Egger regression and MR.RAPS methods, as shown in Supplementary Table 2, Figure 1 and Supplementary Figure 2.

### Pleiotropy and sensitivity analysis

The MR-Egger regression results showed that the horizontal pleiotropy would not bias the causal effect of LTL on FN-BMD (intercept=-0.006, P=0.755), LS-BMD (intercept=-0.003, P=0.874), FA-BMD (intercept=0.007, p=0.826), heel estimated BMD (intercept=-0.003, P=0.652), TB-BMD (intercept=0.011, P=0.548) and TB-BMD (age over 60) (intercept=-0.009, P=0.641) Supplementary Table 2.

**Figure 2:**
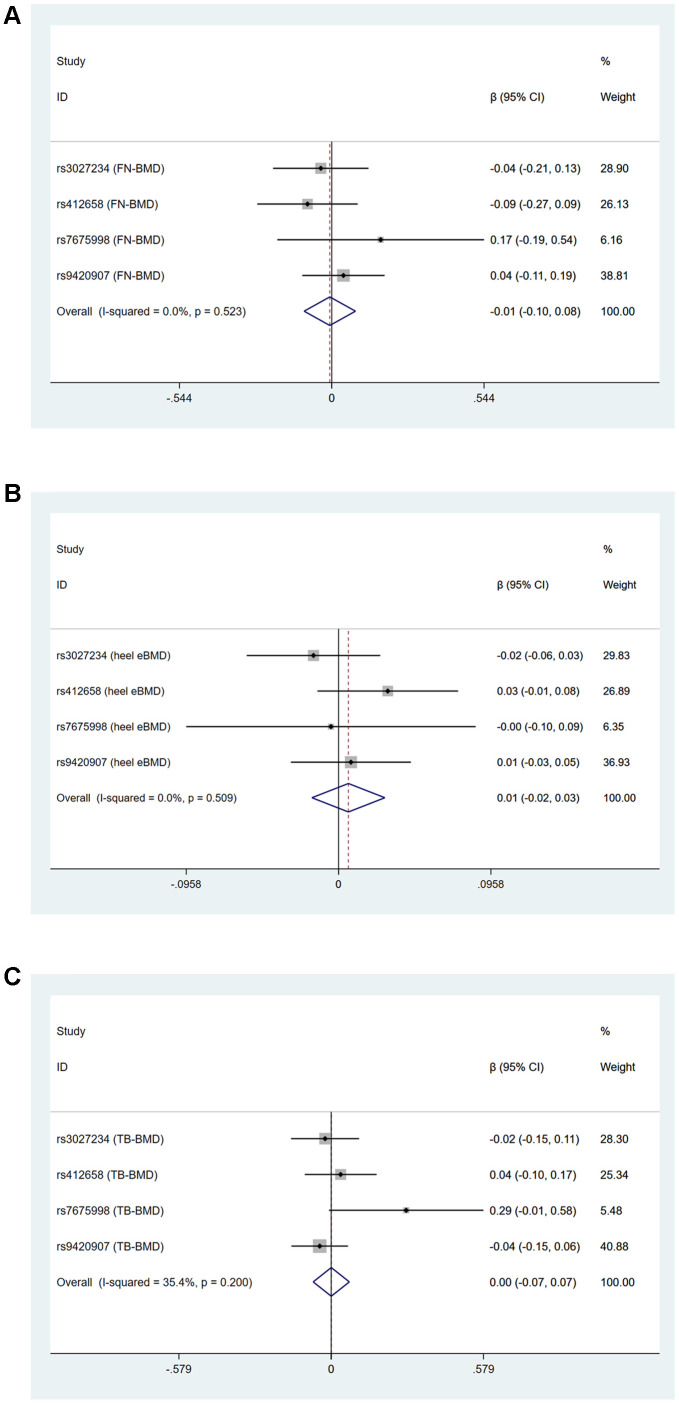
Forest plots for Mendelian randomization estimates of the association of leukocyte telomere length on (**A**) FN-BMD (**B**) heel estimated BMD and (**C**) TB-BMD after excluding rs6028466 (IVW method).

Cochran’s statistics test and I^2^ statistics showed that there was not statistically significant heterogeneity among the effects of individual LTL-associated SNPs on FN-BMD, LS-BMD, FA-BMD and TB-BMD (age over 60) outcomes. However, obvious heterogeneities existed in the effect of LTL-associated SNPs on heel estimated BMD (IVW: Q=11.27, df=4, I^2^=64.5%, P=0.024, MR-Egger: Q=10.41, df=3, P=0.015) and TB-BMD (IVW: Q=9.525, df=4, I^2^=58.0%, P=0.049, MR-Egger: Q=8.268, df=3, P=0.041) Supplementary Table 2 and Figure 1. Because rs6028466 was nominally associated with a reduced level of FN-BMD, heel estimated BMD and TB-BMD. We conducted MR analysis after excluding rs6028466. After removal of this SNP, we observed the causal link between LTL and FN-BMD, heel estimated BMD and TB-BMD was not significant and the heterogeneities vanished Supplementary Table 3 and Figure 2.

In the sensitivity analyses, we observed a consistent no causal association between genetically predicted LTL and level of BMD using the “leave-one-out” method. It suggested the stability of our results Supplementary Figure 3.

## DISCUSSION

Previous publications reported that the alterations of telomere length as well as telomere dysfunction played important roles in age-related impaired homeostasis of bone cells which promoted osteoporosis [18]. In this first two-sample MR analysis assessing the causal link of LTL with BMD, we failed to determine a causal effect of genetically predicted LTL on BMD, which was consistent with some observational studies [25, 26]. In some other publications, they found that shortened LTL was correlated with low BMD and the presence of osteoporosis in women or in the elderly [22–24]. We were not able to conduct separate analyses for men and women, since the sex-stratified GWAS summary data on BMD was not sufficient for performing MR analysis [32]. To prevent identification of individuals from summary results, some information of SNPs on sex-stratified BMD was not included in files. To validate whether there existed a causal effect of genetically predicted TLT on BMD in the elderly, we conducted two-sample MR analysis for age over 60 and did not found a significant causal link between TLT and the level TB-BMD.

In the two-sample MR analysis, we selected SNPs with genome-wide significance and independent inheritance (without any LD) as IVs to detect the causal link between TLT and BMD. After excluding the SNPs associated with phenotypes related to BMD, there were 5 genetic variants finally included as instrumental variables for further MR analysis. F statistic used to assess the instrument-exposure association was much greater than 10, hinting the small possibility of weak instrumental variables bias [33]. To make our conclusion more reliable, we utilized four methods of MR analysis with GWAS summary data of DXA-derived FN-BMD, LS-BMD, FA-BMD, TB-BMD, TB-BMD (age over 60) and ultrasound-derived heel estimated BMD, finding a consistent no causal association between genetically predicted LTL and level of BMD. After removal of rs6028466, which was nominally associated with level of FN-BMD, heel estimated BMD and TB-BMD, MR analysis drew the same conclusion, and the heterogeneities vanished. The MR-Egger regression results showed no horizontal pleiotropy in our analysis. “Leave-one-out” analysis where the MR analysis was performed to assess the influence of each SNP on the overall result indicated our conclusion can be considered robust and convincing.

Our research was the first MR analysis on this topic, it contained several important strengths. First, the causal association of LTL and BMD was not distorted by many confounding factors. It was mitigated by using genetic variants as proxies in MR analysis. Second, four methods of MR analysis on six groups of BMD GWAS summary data were performed to draw a conclusion. Third, to avoid pleiotropic effects, we eliminated SNPs which was recognized associated with confounding factors through searching the Phenoscanner database and no pleiotropic effects was detected by MR-Egger regression method. Fourth, sensitivity analysis was performed and we found the conclusion was of stability. Last, to reduce potential bias, the GWAS summary data we drew for LTL and BMDs was from European descent individuals (except for TB-BMD (age over 60): 86% European ancestry) and adjusted for many common-seen factors, such as: sex, age, height and weight. However, some limitations of our MR analysis need to be considered. First, although selected SNPs were significantly associated with LTL in their respective GWASs, yet 2 SNPs (rs7675998 and rs6028466) did not achieve genome-wide significance with TLT in Mangino et al. study, from which we acquired summary data [31]. Besides, the number of SNPs selected as IVs was limited, raising the risk that they were lack of association with TLT. However, F statistic helped rule out this possibility. We also utilized MR.RAPS method in main analysis, it could give a robust inference for Mendelian randomization with many weak instruments, and it received consistent results with other MR analysis methods. In future updated MR analysis, it is warranted to validate our findings when more and better IVs for LTL become available. Second, through searching Phenoscanner database, information on pleiotropic effects for some of the included SNPs was not available, which left the possibility of pleiotropic effects that had not yet been identified. However, no evidence for the presence of pleiotropy was found, as indicated by the MR-Egger intercept test. Third, the exposure and outcome studies used in two-sample MR analysis should not involve overlapping participants. We were not able to estimate the degree of overlap in the study. However, bias from sample overlap can be minimized by using strong instruments (e.g. F statistic much greater than 10) [34]. Fourth, GWAS summary data of telomere length was from leukocyte, but not the bone tissues. Although telomere length of leukocyte is highly correlated with telomere length in other tissues [21]. Fifth, some observational studies found the association between TLT and BMD in women [22, 23]. And there was a positive correlation of the circulating estradiol with LTL [35]. Estrogen can activate telomerase activity through directly binding to the promoter region of hTERT and prevent telomere shortening, cellular senescence and aging [36]. It suggested the estrogen regulation of telomeres might be linked to level of BMD. However, we were not able to conduct separate MR analyses for women to test our hypothesis since we did not have sex-stratified GWAS summary data for BMD. Sixth, the GWAS summary data mainly concerned individuals of European descent, our results might not be fully representative of whole population. So, we should carefully utilize our conclusion in racially and ethnically diverse populations. Seventh, our MR analysis tested a linear shape of association between LTL and BMD, whereas it did not take the possibility of other shapes of association into account.

In this study, we aimed to determine the causal role of telomere length in level of BMD and osteoporosis by using two-sample MR analysis. However, the results of our research did not provide evidence to support our hypothesis. These findings suggested measures to influence telomere length may have no beneficial effects on BMD and telomere length can not be an indicator to monitor bone mineral density. Updated MR analysis is warranted to revalidate our findings when more and better IVs for LTL and sex-stratified GWAS summary data for BMD are available. Moreover, other factors that may play causal roles in osteoporosis still need to be identified and determined to develop strategies for monitoring, preventing and overcoming osteoporosis.

## MATERIALS AND METHODS

### BMD GWAS summary statistics

To obtain a more comprehensive and reliable conclusion of the causal link between telomere length and BMD, we downloaded several publicly available GWAS summary statistics of BMD from the GEnetic Factors for OSteoporosis Consortium (GEFOS: http://www.gefos.org/) or got them from the IEU GWAS database (https://gwas.mrcieu.ac.uk/). Each study included was approved by their institutional ethics review committees, all participants provided written informed consent. Each SNP was tested for association with BMD, adjusting for many common-seen components, such as: sex, age, height and weight etc.

Three separate GWAS summary statistics of European participants’ Femoral Neck bone mineral density (FN-BMD, n=32735), Lumbar Spine bone mineral density (LS-BMD, n=28498), and Forearm bone mineral density (FA-BMD, n=8143) were downloaded from GEFOS, it is the largest GWAS on DXA-measured BMD to date [6].

Summary GWAS data of ultrasound-derived heel estimated BMD on 426,824 European participants (55% female) were got from IEU GWAS database [7].

A meta-analysis comprising 56,284 individuals of European ancestry was performed to investigate the genetic determinants of Total Body-bone mineral density (TB-BMD) [37]. The meta-analyzed effect size estimates were used in this study. The GWAS summary statistics of TB-BMD were downloaded from the GEFOS website.

To validate the causal link of telomere length and BMD in the elderly, GWAS summary statistics of TB-BMD (age over 60) on 22,504 mixed participants were downloaded from the GEFOS website. Most participants were European ancestry (86%) [37].

### Genetic instrumental variables

We used the 16 single-nucleotide polymorphisms (SNPs) previously utilized by Haycock et al. as IVs to investigate the causal relationships between telomere length and specific diseases [30]. A series of quality control steps were conducted to select eligible instrumental SNPs. As a first step, SNPs significantly associated with LTL of Europeans were searched on the GWAS catalog (https://www.ebi.ac.uk/gwas/) using the genome-wide significance threshold 5×10^−8^, including the seven SNPs from the largest GWAS for LTL of Europeans ancestry (Codd et al., 2013) [38]. To supplement the list with additional potential instruments, we also searched and carefully read the original study reports curated by the GWAS catalog to identify eligible instrumental SNPs. At last, we acquired summary data (i.e., allele frequency, beta value, standard error, and P values) for all SNPs identified in previous process from a meta-analysis of GWASs of LTL, involving 9190 participants of European ancestry [31]. Each SNP was tested for association with LTL, adjusting for covariates, such as: sex and age etc.

### SNP validation

In a standard two-sample MR study, it is important to ensure that the instrumental SNPs for the exposure are not in linkage disequilibrium (LD), since instrumental SNPs in strong LD may cause biased results. In this study, we performed the clumping process with the European samples from the 1000 genomes project to estimate LD between SNPs. The SNPs would be extracted from 1000 genomes data, and LD calculated between them. Amongst those pairs of SNPs that had LD R-square above the specified threshold (R-square = 0.001), only the SNP with the lower P-value would be retained. In the clumping process, we set the window size = 10,000 kb.

According to the assumptions of MR analysis, the selected instrumental SNPs should affect the outcome only through the exposure and not via other biological pathways (i.e., no horizontal pleiotropic effect exist). To explore potential violations of this assumption, each instrumental SNPs was queried against the Phenoscanner database (http://www.phenoscanner.medschl.cam.ac.uk/). When a SNP was associated with another phenotype other than telomere length, we checked whether the associated phenotype was associated with BMD by conducting a literature search. A SNP was excluded from the analysis if it was significantly associated with any phenotype which was a risk factor for osteoporosis or low BMD after Bonferroni correction (p < 0.05 / N, N = the number of SNPs queried). Factors significantly associated with osteoporosis or BMD include rheumatoid arthritis, celiac disease, mean corpuscular hemoglobin, red blood cell count, body fat percentage, premature menopause, cognitive impairment and anemia etc. [39]. By default, if a particular requested SNP is not present in the outcome GWAS then a SNP (proxy) that is in LD with the requested SNP (target) will be searched for instead. LD proxies are defined using 1000 genomes European sample data. The effect of the proxy SNP on the outcome is returned, along with the proxy SNP, the effect allele of the proxy SNP, and the corresponding allele (in phase) for the target SNP. To test whether there was weak instrumental variables bias, namely whether genetic variants selected as instrumental variables had weak association with exposure, we calculated F statistic (F=R^2^(n-k-1)/k(1-R^2^), R^2^: variance of exposure explained by selected instrumental variables, n: sample size, k: number of instrumental variables). If F statistic was much greater than 10 for the instrument-exposure association, the possibility of weak instrumental variables bias was small [33].

### Mendelian randomization estimates

We harmonized the exposure and outcome data to ensure that the effect of a SNP on the exposure, and the effect of that same SNP on the outcome, correspond to the same allele. We combined the summary statistics (β coefficients and standard errors) to estimate the causal associations between LTL and BMD using four methods, which included inverse variance weighting (IVW), weighted median (WM), MR-Egger regression and Robust Adjusted Profile Score (MR.RAPS) method. The IVW method with fixed effect used a meta-analysis approach combining Wald estimates for each SNP (i.e., the β coefficient of the SNP for BMD divided by the β coefficient of the SNP for LTL) to get the overall estimates of the effect of LTL on BMD [40]. If significant heterogeneity was observed, a random-effect model was applied. A WM method might provide correct estimates of the causal effect even when up to 50% of SNPs were invalid IVs (e.g., due to pleiotropy) [41]. MR-Egger regression, basing on the assumption that the pleiotropic associations were independent, performed a weighted linear regression of the outcome coefficients on the exposure coefficients. Egger's test gave a valid test of the null causal hypothesis and a consistent causal effect estimate even when all the genetic variants were invalid IVs [42]. However, MR-Egger estimates might be inaccurate and could be strongly influenced by outlying genetic variants. The WM estimate has been confirmed to have distinct superiorities over MR-Egger for its improved power of causal effect detection and lower type I error [41]. Since we included many weak instrumental variables in the analyses, we carried out a recently proposed method called MR.RAPS to make our results more reliable [43]. This method could give a robust inference for MR analysis with many weak instruments.

### Pleiotropy and sensitivity analysis

We conducted the MR-Egger regression to assess the potential pleiotropic effects of the SNPs used as IVs. The intercept term in MR Egger regression could be a useful indication of whether directional horizontal pleiotropy was driving the results of a MR analysis [44]. We used IVW method and MR-Egger regression to detect heterogeneity. The heterogeneities were quantified by Cochran Q statistics and I^2^ statistics to identify whether there was a higher heterogeneity between causal effects estimated using the variants individually than that would be expected by chance. In our analysis, a P-value of <0.05 or an I^2^ value of >50% would be regarded as significant heterogeneity. Additionally, we performed sensitivity analyses to evaluate the reliability of the association between genetically predicted LTL and risk of low BMD. We performed “leave-one-out” validation analyses, where the MR was performed again but leaving out each SNP in turn, to identify if a single SNP was driving the association.

### Ethics

All data sources were de-identified and publicly available, and thus, no ethical committee approval was required.

**Figure 3:**
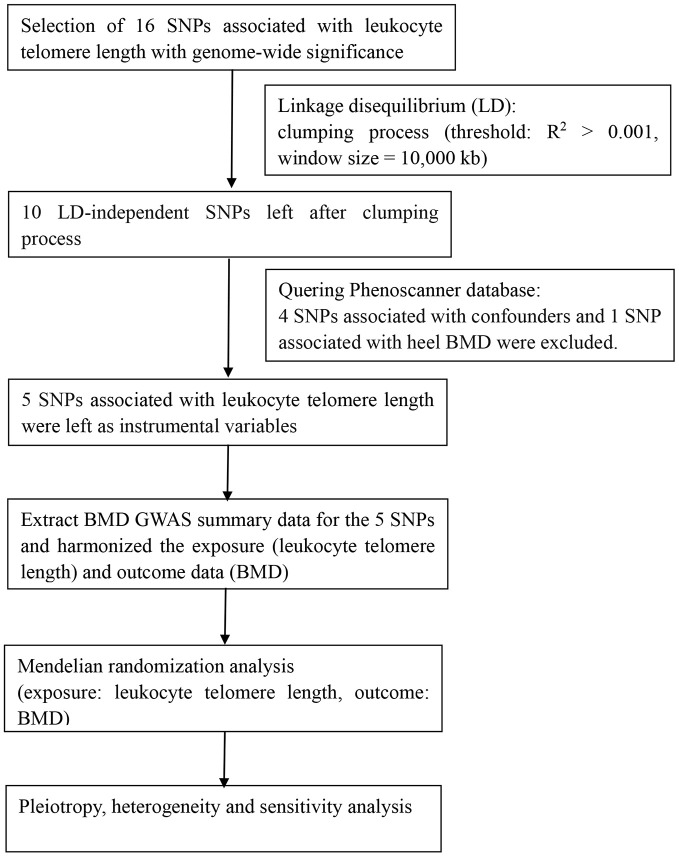
**Flow chart about the analytical methods and process of two-sample MR analysis.**

All statistical analyses were conducted using R version 3.6.3 (R Foundation for Statistical Computing, Vienna, Austria), the Two-Sample MR package [45] and STATA 15 software (Stata Corporation, College Station, TX, USA). P-values <0.05 were considered statistically significant, unless otherwise noted. A flow chart about the analytical methods and how the MR analysis was performed step-by-step was shown in Figure 3.

### Availability of data and materials

All data generated or analyzed during this study are included in this published article and its supplementary information files

## Supplementary Material

Supplementary Figures

Supplementary Table 1

Supplementary Table 2

Supplementary Table 3
