# Supplementary material for: Mendelian randomization study of telomere length and bone mineral density
Source: Aging (Albany NY). 2020 Dec 15;13(2):2015–30. doi: 10.18632/aging.202197 (PMC7880394; doi:10.18632/aging.202197)
Supplement: Supplementary Figures [file aging-13-202197-s001.pdf]

SUPPLEMENTARY FIGURES

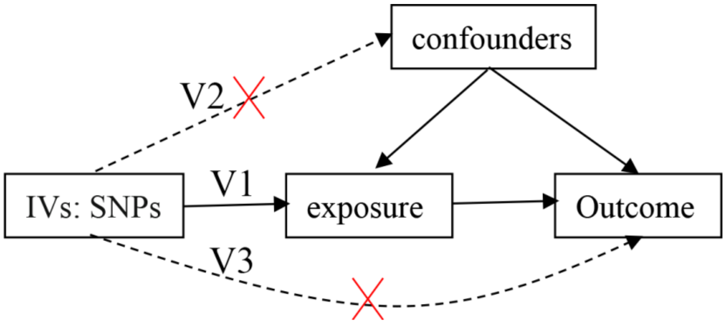

Supplementary Figure 1. The assumptions of the two-sample MR analysis.

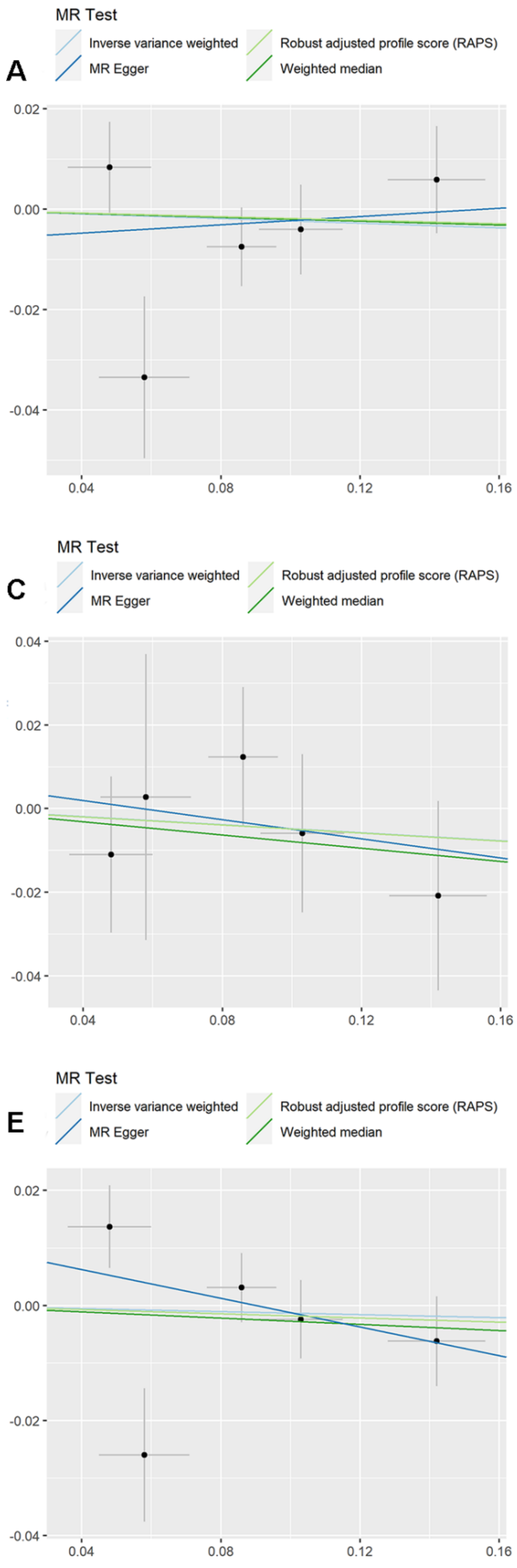

Supplementary Figure 2. Scatter plots for Mendelian randomization analyses of the association of leukocyte telomere length on BMDs (**A**) FN-BMD (**B**) LS-BMD (**C**) FA-BMD (**D**) heel estimated BMD (**E**) TB-BMD and (**F**) TB-BMD (age over 60). Analyses were conducted using the conventional IVW, WM, MR-Egger and MR.RAPS methods. The slope of each line corresponding to estimated MR effect per method.

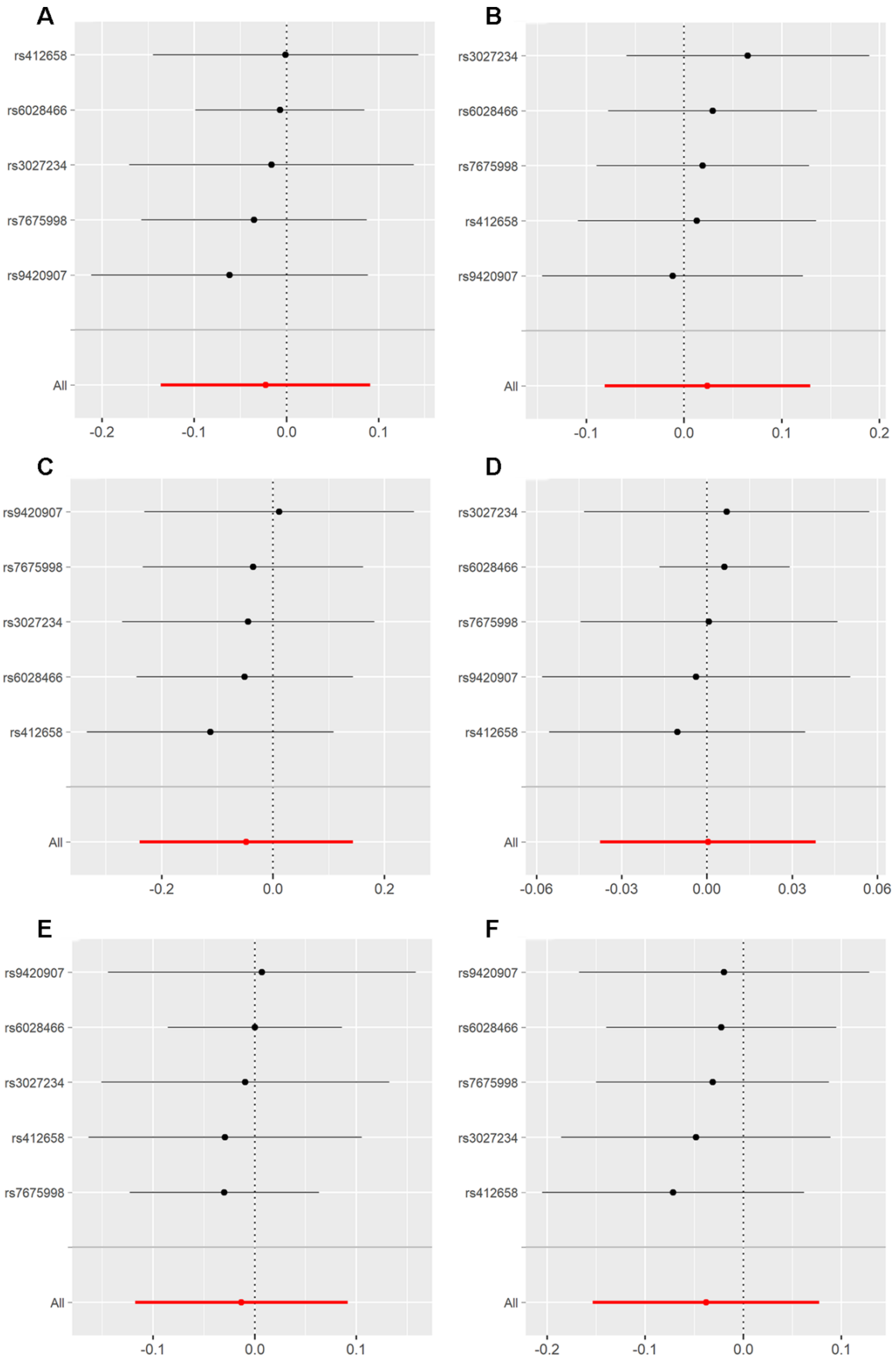

Supplementary Figure 3. "Leave-one-out" analyses for Mendelian randomization analyses of the association of leukocyte telomere length on BMDs (**A**) FN-BMD (**B**) LS-BMD (**C**) FA-BMD (**D**) heel estimated BMD (**E**) TB-BMD and (**F**) TB-BMD (age over 60).
