# Supplementary material for: Mendelian randomization study of telomere length and bone mineral density
Source: Aging (Albany NY). 2020 Dec 15;13(2):2015–30. doi: 10.18632/aging.202197 (PMC7880394; doi:10.18632/aging.202197)
Supplement: Supplementary Table 1 [file aging-13-202197-s002.docx]

Supplementary Table 1. The genetic instruments for Mendelian randomization analysis of leukocyte telomere length (exposure) and BMDs (outcome).

| SNP | Association with leukocyte telomere length | | |  | Association with FN-BMD | |  | Association with LS-BMD | |  | Association with FA-BMD | |  | Association with heel estimated BMD | |  | Association with TB-BMD | |  | Association with TB-BMD (age over 60) | |
| --- | --- | --- | --- | --- | --- | --- | --- | --- | --- | --- | --- | --- | --- | --- | --- | --- | --- | --- | --- | --- | --- |
|  | β(Se) | P value^1^ | P value^2^ |  | β(Se) | P value |  | β(Se) | P value |  | β(Se) | P value |  | β(Se) | P value |  | β(Se) | P value |  | β(Se) | P value |
| rs7675998 | 0.048(0.012) | 1.00e-2 | 4.35e-16 |  | 0.008 (0.009) | 0.364 |  | 0.005 (0.011) | 0.662 |  | -0.011 (0.019) | 0.564 |  | -0.0002 (0.002) | 0.460 |  | 0.014 (0.007) | 0.056 |  | -0.008 (0.012) | 0.537 |
| rs9420907 | 0.142(0.014) | 1.14e-11 | 7.00e-11 |  | 0.006 (0.011) | 0.587 |  | 0.012 (0.012) | 0.355 |  | -0.021 (0.023) | 0.368 |  | 0.001 (0.003) | 0.490 |  | -0.006 (0.008) | 0.428 |  | -0.010 (0.013) | 0.477 |
| rs3027234 | 0.103(0.012) | 2.75e-8 | 2.00e-8 |  | -0.004 (0.009) | 0.660 |  | -0.008 (0.010) | 0.433 |  | -0.006 (0.019) | 0.759 |  | -0.002 (0.002) | 0.200 |  | -0.002 (0.007) | 0.722 |  | -0.001 (0.012) | 0.922 |
| rs412658 | 0.086(0.010) | 1.83e-8 | 1.00e-8 |  | -0.007 (0.008) | 0.354 |  | 0.005 (0.009) | 0.610 |  | 0.012 (0.017) | 0.470 |  | 0.003 (0.002) | 0.140 |  | 0.003 (0.006) | 0.607 |  | 0.005 (0.010) | 0.602 |
| rs6028466 | 0.058(0.013) | 4.00e-3 | 2.57e-8 |  | -0.033 (0.016) | 0.042 |  | -0.010 (0.019) | 0.623 |  | 0.003 (0.034) | 0.937 |  | -0.012 (0.004) | 0.007 |  | -0.026 (0.012) | 0.025 |  | -0.030 (0.019) | 0.120 |

Notes: β:effect size estimates; Se: standard error; P value^1^: P value from summary data of Mangino et al.[38]; P value^2^: P value from original study reports curated by the GWAS catalog. FN-BMD: Femoral Neck bone mineral density, LS-BMD: Lumbar Spine bone mineral density, FA-BMD: Forearm bone mineral density, TB-BMD: Total Body-bone mineral density.
