# Supplementary material for: Mendelian randomization study of telomere length and bone mineral density
Source: Aging (Albany NY). 2020 Dec 15;13(2):2015–30. doi: 10.18632/aging.202197 (PMC7880394; doi:10.18632/aging.202197)
Supplement: Supplementary Table 2 [file aging-13-202197-s003.docx]

**Table 2.** **Mendelian randomization estimates of the association of leukocyte telomere length on BMDs.**

| BMDs | IVW | | | | |  | WMM | |  | MR-Egger | | | |  |  |  | MR.RAPS | |
| --- | --- | --- | --- | --- | --- | --- | --- | --- | --- | --- | --- | --- | --- | --- | --- | --- | --- | --- |
|  | β(95%CI) | P value | Cochran Q statistics (df) | I^2^ | P value |  | β(Se) | P value |  | Slope(Se) | P value | Intercept(Se) | P value | Cochran Q statistics (df) | P value |  | β(Se) | P value |
| FN-BMD | -0.023 (-0.113,0.068) | 0.695 | 6.327 (4) | 36.8% | 0.176 |  | -0.019 (0.055) | 0.724 |  | 0.041 (0.198) | 0.849 | -0.006 (0.019) | 0.755 | 6.091 (3) | 0.107 |  | -0.018 (0.048) | 0.711 |
| LS-BMD | 0.024(-0.081,0.129) | 0.657 | 2.071 (4) | 0.0% | 0.723 |  | 0.061 (0.068) | 0.367 |  | 0.050 (0.162) | 0.777 | -0.003 (0.015) | 0.874 | 2.041 (3) | 0.564 |  | 0.024 (0.056) | 0.669 |
| FA-BMD | -0.048 (-0.240,0.143) | 0.622 | 1.593 (4) | 0.0% | 0.810 |  | -0.079 (0.123) | 0.521 |  | -0.114 (0.293) | 0.722 | 0.007 (0.027) | 0.826 | 1.536 (3) | 0.674 |  | -0.048 (0.103) | 0.636 |
| heel estimated BMD | -0.010 (-0.053,0.032) | 0.986 | 11.27 (4) | 64.5% | 0.024 |  | 0.004 (0.015) | 0.769 |  | 0.031 (0.065) | 0.667 | -0.003 (0.006) | 0.652 | 10.41 (3) | 0.015 |  | 0.003 (0.013) | 0.806 |
| TB-BMD | -0.010 (-0.128,0.108) | 0.806 | 9.525 (4) | 58.0% | 0.049 |  | -0.027 (0.042) | 0.512 |  | -0.125 (0.175) | 0.528 | 0.011 (0.017) | 0.548 | 8.268 (3) | 0.041 |  | -0.018 (0.050) | 0.721 |
| TB-BMD (age over 60) | -0.038 (-0.154,0.077) | 0.518 | 3.163 (4) | 0.0% | 0.531 |  | -0.029 (0.067) | 0.663 |  | 0.049 (0.179) | 0.801 | -0.009 (0.017) | 0.641 | 2.896 (3) | 0.408 |  | -0.037 (0.062) | 0.545 |

**Notes:** IVW: inverse variance weighting, WM: weighted median, MR.RAPS: Robust Adjusted Profile Score.
