# Supplementary material for: Mendelian randomization study of telomere length and bone mineral density
Source: Aging (Albany NY). 2020 Dec 15;13(2):2015–30. doi: 10.18632/aging.202197 (PMC7880394; doi:10.18632/aging.202197)
Supplement: Supplementary Table 3 [file aging-13-202197-s004.docx]

**Supplementary Table 3. Mendelian randomization estimates** **of the association of leukocyte telomere length and FN-BMD, heel estimated BMD and TB-BMD after excluding** **rs6028466**

.

| BMDs | IVW | | | | |  | WMM | |  | MR-Egger | | | | | |  | MR.RAPS | |
| --- | --- | --- | --- | --- | --- | --- | --- | --- | --- | --- | --- | --- | --- | --- | --- | --- | --- | --- |
|  | β(95%CI) | P value | Cochran Q statistics (df) | I^2^ | P value |  | β(Se) | P value |  | Slope(Se) | P value | intercept(Se) | P value | Cochran Q statistics (df) | P value |  | β(Se) | P value |
| FN-BMD | -0.007(-0.099,0.085 ) | 0.880 | 2.244 (3) | 0.0% | 0.523 |  | -0.017 (0.056) | 0.766 |  | -0.037 (0.152) | 0.831 | 0.003 (0.015) | 0.855 | 2.197 (2) | 0.333 |  | -0.007 (0.049) | 0.885 |
| heel estimated BMD | 0.006 (-0.017,0.029) | 0.598 | 2.317 (3) | 0.0% | 0.509 |  | 0.005 (0.015) | 0.742 |  | 0.003 (0.039) | 0.943 | 0.0003 (0.004) | 0.942 | 2.310 (2) | 0.315 |  | 0.006 (0.012) | 0.611 |
| TB-BMD | 0.0003 (-0.068,0.069) | 0.994 | 4.644 (3) | 35.4% | 0.200 |  | -0.027 (0.043) | 0.533 |  | -0.218 (0.110) | 0.187 | 0.023 (0.011) | 0.172 | 0.278 (2) | 0.870 |  | -0.005 (0.038) | 0.903 |

**Notes:** IVW: inverse variance weighting, WM: weighted median, MR.RAPS: Robust Adjusted Profile Score.
